# LOXL2-dependent deacetylation of aldolase A induces metabolic reprogramming and tumor progression^☆^

**DOI:** 10.1016/j.redox.2022.102496

**Published:** 2022-10-03

**Authors:** Ji-Wei Jiao, Xiu-Hui Zhan, Juan-Juan Wang, Li-Xia He, Zhen-Chang Guo, Xiu-E Xu, Lian-Di Liao, Xin Huang, Bing Wen, Yi-Wei Xu, Hai Hu, Gera Neufeld, Zhi-Jie Chang, Kai Zhang, Li-Yan Xu, En-Min Li

**Affiliations:** a The Key Laboratory of Molecular Biology for High Cancer Incidence Coastal Chaoshan Area, Department of Biochemistry and Molecular Biology, Shantou University Medical College, Shantou, 515041, China; b Department of Orthopedics, Research Center of Translational Medicine, The Second Affiliated Hospital of Shantou University Medical College, Shantou, 515041, China; c Guangdong Provincial Key Laboratory of Infectious Diseases and Molecular Immunopathology, Institute of Oncologic Pathology, Shantou University Medical College, Shantou, 515041, China; d Department of Biochemistry and Molecular Biology, Tianjin Medical University, Tianjin, 300070, China; e Department of Clinical Laboratory, The First Affiliated Hospital of Shantou University Medical College, Shantou, 515041, China; f Department of Clinical Laboratory Medicine, Cancer Hospital of Shantou University Medical College, Shantou, 515041, China; g Department of Oncology, Sun Yat-Sen Memorial Hospital, Sun Yat-Sen University, Guangzhou, 510120, China; h Technion Integrated Cancer Center, The Bruce Rappaport Faculty of Medicine, Technion, Israel Institute of Technology, Haifa, 31096, Israel; i State Key Laboratory of Membrane Biology, School of Medicine, National Engineering Laboratory for Anti-tumor Therapeutics, Tsinghua University, Beijing, 10084, China

**Keywords:** Lysyl oxidase-like 2, Aldolase, Glycolysis, Deacetylation, Tumorigenesis

## Abstract

Lysyl-oxidase like-2 (LOXL2) regulates extracellular matrix remodeling and promotes tumor invasion and metastasis. Altered metabolism is a core hallmark of cancer, however, it remains unclear whether and how LOXL2 contributes to tumor metabolism. Here, we found that LOXL2 and its catalytically inactive L2Δ13 splice variant boost glucose metabolism of esophageal tumor cells, facilitate tumor cell proliferation and promote tumor development *in vivo*. Consistently, integrated transcriptomic and metabolomic analysis of a knock-in mouse model expressing L2Δ13 gene revealed that LOXL2/L2Δ13 overexpression perturbs glucose and lipid metabolism. Mechanistically, we identified aldolase A, glyceraldehyde-3-phosphate dehydrogenase and enolase as glycolytic proteins that interact physically with LOXL2 and L2Δ13. In the case of aldolase A, LOXL2/L2Δ13 stimulated its mobilization from the actin cytoskeleton to enhance aldolase activity during malignant transformation. Using stable isotope labeling of amino acids in cell culture (SILAC) followed by proteomic analysis, we identified LOXL2 and L2Δ13 as novel deacetylases that trigger metabolic reprogramming. Both LOXL2 and L2Δ13 directly catalyzed the deacetylation of aldolase A at K13, resulting in enhanced glycolysis which subsequently reprogramed tumor metabolism and promoted tumor progression. High level expression of LOXL2/L2Δ13 combined with decreased acetylation of aldolase-K13 predicted poor clinical outcome in patients with esophageal cancer. In summary, we have characterized a novel molecular mechanism that mediates the pro-tumorigenic activity of LOXL2 independently of its classical amine oxidase activity. These findings may enable the future development of therapeutic agents targeting the metabolic machinery via LOXL2 or L2Δ13.

**Highlight of the study:**

LOXL2 and its catalytically inactive isoform L2Δ13 function as new deacetylases to promote metabolic reprogramming and tumor progression in esophageal cancer by directly activating glycolytic enzymes such as aldolase A.

## 1 Introduction

The lysyl oxidase (LOX) family is comprised of five members (LOX and LOXL1-4) whose primary function is to oxidize the ε-amino group of peptidyl lysine residues to aldehyde residues, thereby covalently crosslinking elastin and/or collagens in the extracellular matrix (ECM) [1,2]. The ECM remodeling provides structural scaffolding and contextual information for cells, which is required for both tissue integrity and expansion. Aberrant expression and activity of lysyl oxidase-like 2 (LOXL2) as a secreted copper- and quinone-dependent enzyme is involved in pathological processes predominantly associated with the ECM remodeling, such as Wilson's disease, heart failure and cholestasis [[3], [4], [5]]. LOXL2-induced alterations of ECM organization are associated with the induction of abnormal fibrosis, which in tumors promotes tumor cell invasiveness and tumor metastasis [[6], [7], [8]]. Moreover, elevated expression of LOXL2 as a result of hypoxic stress has been found to modulate gene transcription and to promote epithelial-to-mesenchymal transition (EMT), contributing to the enhancement of tumor cell motility, invasiveness and metastasis [9,10]. It has also been reported that specific catalytically inactive mutants of LOXL2 mediate keratinocyte differentiation and EMT caused by an interaction with the transcription factor Snail1 [[11], [12], [13]]. However, the contribution of the non-enzymatic functions of LOXL2 to tumor progression has not yet been studied in depth.

Changes in the organization of the ECM can influence cellular metabolism [14]. For example, degradation of ECM-associated hyaluronic acid can affect glucose uptake and enhance glycolysis [15]. LOXL2 is also present in intra-cellular compartments, such as the nucleus and cytoplasm of cells [9,16,17], suggesting that LOXL2 as well as additional lysyl oxidases perhaps also affects metabolism through interacting with intracellular proteins. Although elevated expression of lysyl oxidases and metabolic reprogramming are coincident in multiple biological contexts [18,19], their underlying mechanistic links are not well established.

We have previously identified a novel LOXL2 splice variant L2Δ13 (mRNA, GenBank accession number KF928961; protein, GenBank accession number AHJ59530) in human esophageal squamous cell carcinoma (hereinafter referred to esophageal cancer) cells [20]. The L2Δ13 isoform was also observed in immortalized esophageal epithelial cells and in various types of cancer cells [20]. Unlike extracellular full-length LOXL2, L2Δ13 lacks exon 13, which is located in the highly conserved catalytic C-terminal domain of LOXL2, resulting in the complete loss of amine oxidase activity. In addition, L2Δ13 fails to be secreted from cells. Thus, L2Δ13 represents a LOXL2 form that promotes certain biological activities independent of the classical enzymatic function of the lysyl oxidases. High level expression of full-length LOXL2, as well as its L2Δ13 isoform, promotes cell migration and invasion of esophageal cancer cells *in vitro* and *in vivo*, which is further linked to tumor metastasis and poor clinical outcome of esophageal cancer patients [16,21]. In this study, we find that L2Δ13 and LOXL2 physically interact with and simultaneously deacetylate glycolytic enzymes, such as aldolase A, to facilitate glucose metabolism, suggesting that LOXL2/L2Δ13 induces metabolic alterations which in turn may be responsible for part of the pro-tumorigenic effects of LOXL2.

## 2 Results

### 2.1 Full-length LOXL2 and L2Δ13 enhance esophageal cancer cell proliferation *in vitr*o and *in vivo*

**Fig. 1:**
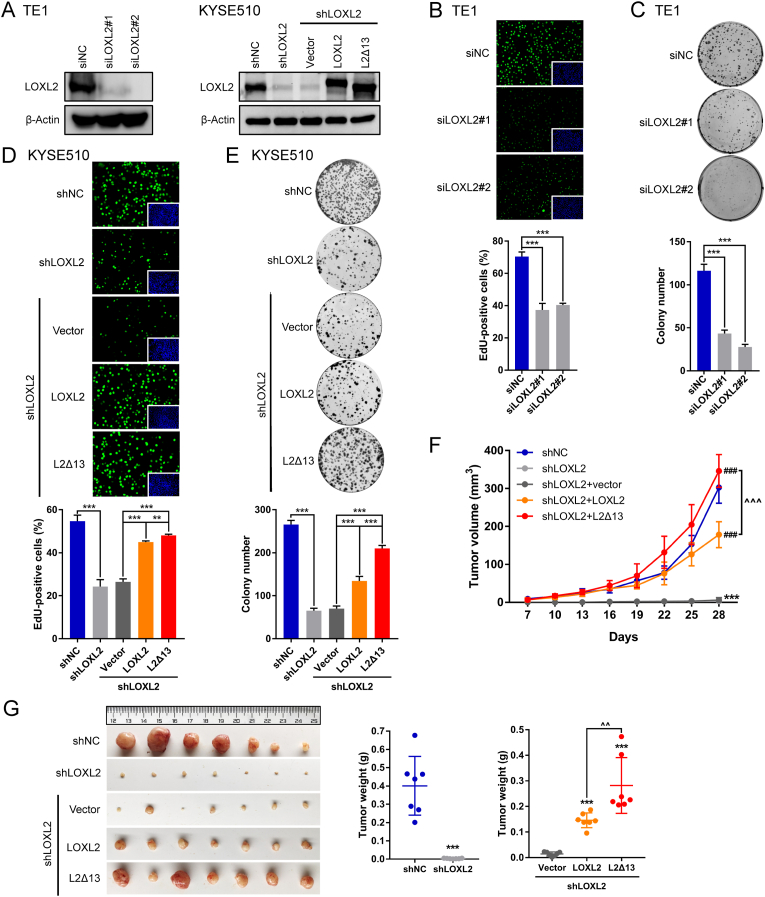
Full-length LOXL2 and L2Δ13 promote tumor cell proliferation *in vitro* and *in vivo*. **(A)** Western blotting assays of esophageal cancer cells following depletion of LOXL2 by specific siRNAs (left) and LOXL2 stably-depleted esophageal cancer cells following ectopic re-expression of LOXL2 or L2Δ13 (right). **(B and C)** EdU **(B)** and colony formation **(C)** assays of esophageal cancer cells following silencing of LOXL2. Shown are representative images of EdU-labeled cells (green) and nucleus stained by Hoechst (blue). Error bars indicate mean ± SD; n = 5 for EdU, n = 3 for colony formation. ***P < 0.001. **(D and E)** EdU **(D)** and colony formation **(E)** assays for KYSE510 cells infected with a scrambled shRNA or shLOXL2 and LOXL2-silenced KYSE510 cells expressing empty vector, LOXL2-Flag or L2Δ13-Flag. Error bars represent mean ± SD of triplicates. **P < 0.01 or ***P < 0.001. **(F)** Indicated KYSE510 stably-infected cells (1 × 10^6^ in 100 μL PBS) were implanted subcutaneously into nude mice. Tumor volumes of xenograft mice were measured at the indicated time points, and tumors were excised after 30 days (n = 7). *Different from xenografts injected KYSE510 cells infected with shNC, ***P < 0.001; ^#^different from xenografts injected with LOXL2-depleted cells expressing vector, ^###^*P* < 0.001. ^^^^*P* < 0.01 or ^^^^^*P* < 0.001. *P*-values were determined by a *t*-test. **(G)** Images of dissected tumors and summary of average tumor weights measured at end point. (For interpretation of the references to color in this figure legend, the reader is referred to the Web version of this article.)

To study the contribution of either full-length LOXL2 or its spliced isoforms to tumor cell proliferation, we performed loss- and gain-of function assays in esophageal cancer cells. We silenced LOXL2 expression in esophageal cancer cells using specific siRNAs or shRNAs, and then conducted rescue experiments by ectopically re-expressing full-length LOXL2 or L2Δ13 in the LOXL2-silenced cancer cells as previously described (Fig. 1A; Supplementary Fig. S1A) [16]. LOXL2 depletion strikingly inhibited the tumor cell proliferation of the TE1 and KYSE510 cells, as determined by the measurement of DNA synthesis and cell colony formation (Fig. 1B and C; Supplementary Figs. S1B and S1C). LOXL2 depletion also greatly decreased the accumulation of neutral lipids in cell metabolism of these esophageal cancer cells (Supplementary Fig. S1D). In contrast, re-expression of either LOXL2 or L2Δ13 in LOXL2-depleted cells fully rescued their impaired proliferative capacity (Fig. 1D and E). Surprisingly, L2Δ13 seemed more effective than full-length LOXL2 in the colony formation assay (Fig. 1E). These *in vitro* results were further verified by *in vivo* xenograft rescue experiments in which we expressed the cDNAs encoding either LOXL2 or L2Δ13 in KYSE510 cells silenced for LOXL2 expression. The development of subcutaneous tumors from implanted KYSE510 cells silenced for LOXL2 expression was strongly inhibited, whereas re-expression of LOXL2 or L2Δ13 in the LOXL2-silenced cells partially restored tumor development (Fig. 1F and G; Supplementary Fig. S1E). Interestingly, L2Δ13 in this assay also rescued tumor development more efficiently than full-length LOXL2.

### 2.2 LOXL2 and L2Δ13 regulate metabolic reprogramming

**Fig. 2:**
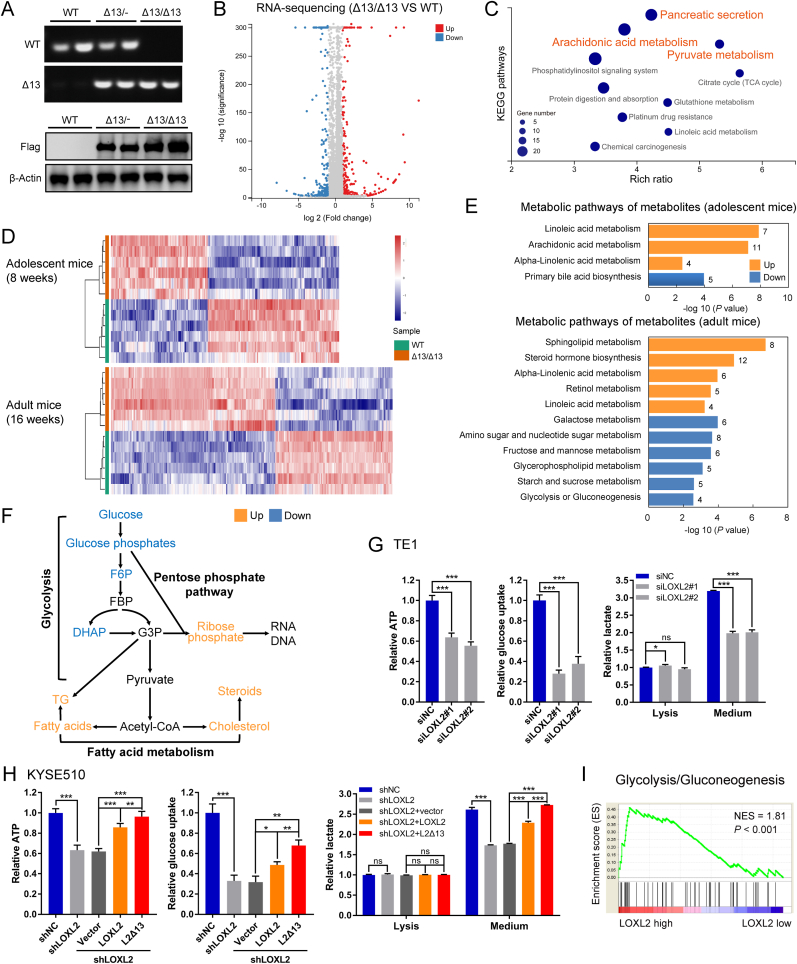
LOXL2 and L2Δ13 regulate metabolic reprogramming. **(A)** Top: PCR conformation of overexpression of L2Δ13 in C57BL/6J mice; bottom: western blotting analysis of livers from wild-type (WT), heterozygous (Δ13/-) and homozygous L2Δ13-mice (Δ13/Δ13). **(B)** Volcano plot of differential gene expression between L2Δ13-overexpressing and age-matched wild-type control mice in RNA-sequencing analysis (n = 6; GSE145238). **(C)** Significant patterns for KEGG pathways of differentially-expressed genes of L2Δ13-overexpressing and age-matched wild-type mice (n = 6; GSE145238). **(D)** Heatmap of metabolite clusters in L2Δ13-overexpressing and paired wild-type mice measured by LC-MS-based metabolomics (n = 6). **(E)** Metabolic pathways analysis of the significantly enriched metabolites (*q* value < 0.05, fold change ≥1.2) using MetaboAnalyst. **(F)** Schematic representation of the L2Δ13-regulated metabolic flux in mice under physiological conditions. F6P, fructose-6-phosphate; FBP, fructose-1,6-bisphosphate; G3P, glyceraldehyde 3-phosphate; Actyl-CoA, actyl coenzyme A. **(G and H)** Levels of ATP, glucose uptake and lactate of esophageal cancer cells following depletion of LOXL2 by specific siRNAs **(G)** and LOXL2-depleted esophageal cancer cells following ectopic re-expression of LOXL2 or L2Δ13 **(H)**. Mean ± SD, n = 3–5. *P < 0.05, **P < 0.01 or ***P < 0.001. ns, not significant. **(I)** Gene set enrichment analysis (GSEA) plot shows that LOXL2 gene expression positively correlated with genes in the glycolysis/gluconeogenesis pathway (KEGG database/hsa00010, gene numbers = 68) in patients with esophageal cancer (NCBI/GEO/GSE23400, n = 53). NES, normalized enrichment score. *P*-values were determined by Pearson's correlation.

Cell growth and proliferation in cancer cells are reported to be linked with altered metabolism [22]. We previously found that LOXL2 predominantly regulated genes involved in several metabolic pathways in esophageal cancer, such as carbohydrate metabolism and lipid metabolism [16]. Using a Cre/LoxP-based gene-targeting strategy, we generated a gain-of-function mouse model to further explore the role of LOXL2/L2Δ13 in metabolic regulation. Flag-tagged human *L2Δ13* cDNA was inserted by homologous recombination into the *ROSA26* locus and expressed under the control of the CAG promoter following Cre-mediated excision (Fig. 2A; Supplementary Fig. S2A). To determine which genes are regulated by L2Δ13 *in vivo*, next generation RNA sequencing was performed to compare the RNA expression profile of liver tissues excised from L2Δ13-overexpressing and wild-type mice (GSE145238). Transcriptomic data identified 599 genes differentially expressed by greater than a 2-fold change in L2Δ13-overexpressing mice relative to paired wild-type samples (Fig. 2B). Importantly, more than 60% of these highly altered genes were involved in diverse metabolic processes, including pyruvate metabolism, TCA cycle, lipid metabolism and amino acid metabolism (Fig. 2C). To determine if these L2Δ13-induced changes in gene expression are reflected by changes in the concentration of metabolites, we measured the levels of metabolites contained in mouse livers from these two groups by liquid chromatography-mass spectrometry (LC-MS) analysis. Consistently, metabolomics data derived from the livers of both adolescent and adult mice showed significant differences in the levels of metabolites between L2Δ13 mice and wild-type mice, further indicating that L2Δ13 induces metabolic reprogramming (Fig. 2D). Metabolomic pathway analysis revealed that L2Δ13 facilitates glycolysis/gluconeogenesis, fructose and mannose metabolism, and amino acid metabolism (Fig. 2E; Supplementary Fig. S2B). Moreover, L2Δ13 contributed to higher lipid metabolism, as illustrated by enrichments in intermediate metabolites of linoleic acid metabolism, sphingolipid metabolism, as well as steroid hormone biosynthesis (Fig. 2E; Supplementary Fig. S2B). These results indicate that L2Δ13 overexpression enhances the expression of enzymes important for glucose consumption and lipid metabolism (Fig. 2F).

To characterize the molecular mechanisms by which LOXL2/L2Δ13 regulates metabolism, we analyzed the metabolic status of esophageal nonmalignant cells and esophageal cancer cells in the presence and absence of LOXL2. Glycolysis, an essential pathway for glucose metabolism, utilizes glucose to generate pyruvate and subsequently ATP and lactate [23]. Intriguingly, ATP levels, along with glucose uptake and lactate production, were enhanced in esophageal epithelial cells overexpressing either LOXL2 or L2Δ13, indicating that LOXL2 and L2Δ13 may facilitate metabolism via enhancement of glycolysis (Supplementary Figs. S3A and S3B). Glucose metabolism shifts from oxidative phosphorylation to aerobic glycolysis in proliferating cancer cells (the Warburg effect) [24]. Consistently, depletion of LOXL2 greatly decreased ATP formation, glucose uptake and lactate buildup in TE1 and KYSE510 esophageal cancer cells (Fig. 2G; Supplementary Fig. S3C). ATP formation was completely rescued and lactate production increased to background levels following ectopic expression of LOXL2 or L2Δ13 (Fig. 2H), suggesting that LOXL2 and L2Δ13 specifically promote glycolysis in cancer cells. Furthermore, microarray data of patients with esophageal cancer also indicated that LOXL2 expression positively correlated with enhanced glycolysis and gluconeogenesis pathways in clinical settings (Fig. 2I). Taken together, these observations suggest that LOXL2/L2Δ13 functions as a novel regulator of metabolic reprogramming.

### 2.3 LOXL2 and L2Δ13 drive glycolysis by interacting with glycolytic proteins

**Fig. 3:**
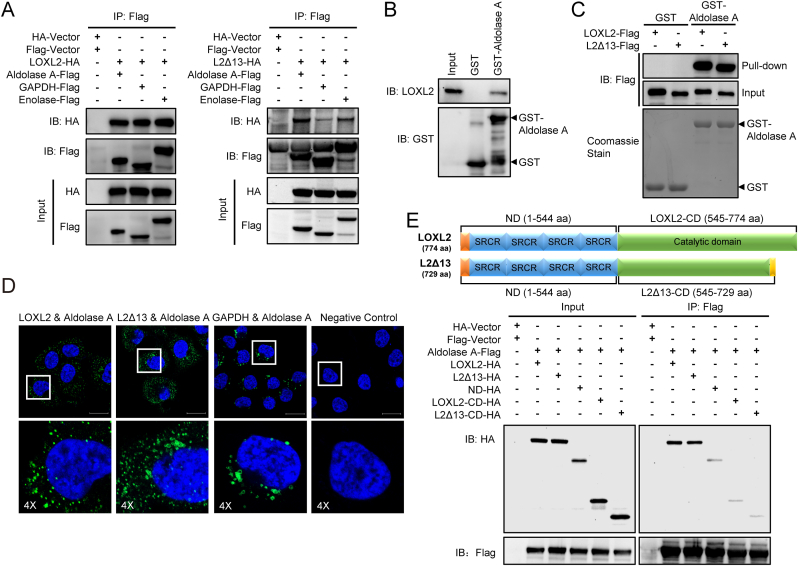
LOXL2 and L2Δ13 regulate glycolysis through interacting with glycolytic enzymes. **(A)** HEK293T cells were transfected with LOXL2-HA or L2Δ13-HA and aldolase A-Flag, GAPDH-Flag, enolase-Flag or empty vectors (Flag-vector and HA-vector). Whole cell lysates were co-immunoprecipitated with anti-Flag and probed by western blotting with the indicated antibodies. **(B)** GST pull-down assay in which either GST-tagged aldolase A or control GST was used to pull down endogenous LOXL2 in whole cell lysates extracted from KYSE510 cells. **(C)** Purified recombinant GST-aldolase A and GST from bacteria were retained on glutathione resins, incubated with either immunopurified LOXL2-Flag or L2Δ13-Flag from HEK293T cells and then immunoblotted with the antibody against Flag. **(D)***In situ* PLA detection of the interaction between LOXL2 and aldolase A, L2Δ13 and aldolase A, and GAPDH with aldolase A in KYSE510 cells that express these proteins endogenously. PLA signals are shown in green, and nuclei are stained blue by DAPI dye. The detection of GAPDH and aldolase A complexes serves as a positive control. The negative control was performed with only anti-aldolase A antibody. Scale bar, 20 μm. **(E)** Top: illustration of the C- and N-terminal domains (CD and ND, respectively) of full-length LOXL2 and L2Δ13; bottom: co-IP assay of aldolase A with different domains of full-length LOXL2 and L2Δ13. (For interpretation of the references to color in this figure legend, the reader is referred to the Web version of this article.)

Our previous liquid chromatography tandem mass spectrometry (LC-MS/MS) analysis of proteins that co-purify with LOXL2 and/or L2Δ13 in esophageal cancer cells identified several known metabolism-associated proteins as interacting partners that interact physically with LOXL2/L2Δ13, including glycolytic enzymes [16]. Co-immunoprecipitation assays validated the interaction of LOXL2 and L2Δ13 with several such enzymes, including aldolase A, glyceraldehyde-3-phosphate dehydrogenase (GAPDH) and enolase (Fig. 3A). Moreover, increased expression of these glycolytic enzymes correlated with poor clinical outcome in esophageal cancer patients (Supplementary Figs. S4A and S4B). A series of glutathione S-transferase (GST) pull down assays further verified that both LOXL2 and L2Δ13 bound strongly to GST-aldolase A but not to GST (Fig. 3B and C and Supplementary Fig. S5A). Confocal immunofluorescence staining indicated that either LOXL2 or L2Δ13 colocalized with aldolase A in esophageal cancer cells which express all of these proteins endogenously (Supplementary Fig. S5B). Importantly, *in situ* proximity ligation assay (PLA) detected LOXL2/L2Δ13-aldolase A complexes in esophageal cancer cells, confirming that this interaction also exists *in vivo* (Fig. 3D). To characterize the domains required for the binding of LOXL2/L2Δ13 to aldolase, we expressed in HEK293T cells a deletion mutant containing the shared N-terminal domain regions of LOXL2 and L2Δ13 (1–544 aa) as well as constructs expressing the C-terminus of LOXL2 (amino-acids 545–774) and L2Δ13 (amino-acids 545–729). The interaction of either LOXL2 or L2Δ13 with aldolase was largely abolished in HEK293T cells expressing each of the deletion mutants of LOXL2 and L2Δ13, suggesting that both the N- and C-terminal domains are required for efficient binding of LOXL2 and L2Δ13 to aldolase (Fig. 3E; Supplementary Fig. S5C). These findings indicate that LOXL2 and L2Δ13 may functionally drive tumor progression through induction of glycolysis as a result of interactions with glycolytic proteins.

### 2.4 LOXL2 and L2Δ13 promote the mobilization and enzymatic activity of aldolase A

**Fig. 4:**
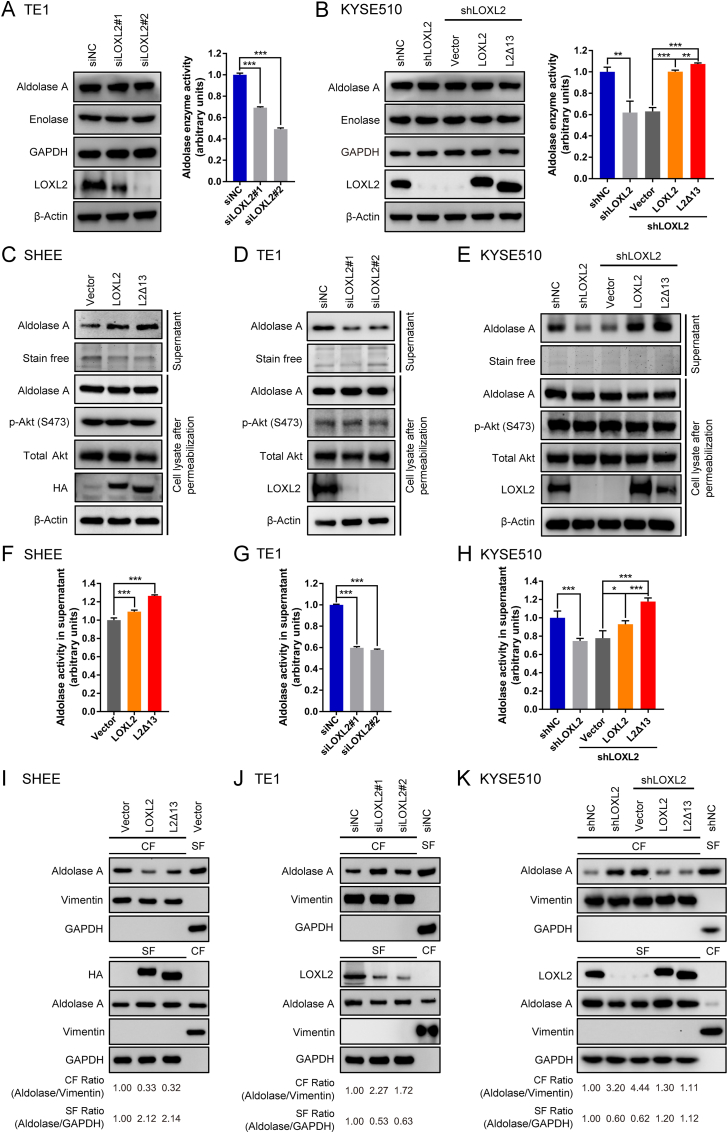
LOXL2 and L2Δ13 promote mobilization of aldolase A and increase its catalytic activity. **(A and B)** Western blotting detection (left) and aldolase enzyme activity assay (right) of esophageal cancer TE1 and KYSE510 cells upon LOXL2 silencing or re-expression of either LOXL2 or L2Δ13. **(C**–**E)** Nonmalignant esophageal epithelial SHEE cells and esophageal cancer cells (TE1 and KYSE510) were permeabilized with digitonin (30 μg/mL) for 5 min. Supernatant (top two panels) and cell lysate (bottom five panels) for each assay were subjected to western blotting as indicated. **(F–H)** Quantification of aldolase activity in the diffusible fraction (supernatant) by immunoblotting of cells in **(C**–**E)**. Bar graphs represent means ± SD, n = 3 or 4. *P < 0.05, **P < 0.01 or ***P < 0.001 by *t*-test. **(I–K)** SHEE, TE1 and KYSE510 cells were lysed and fractionated into cytoskeletal fraction (CF) and soluble fraction (SF). Fractions from indicated cells transfected with empty vector, scrambled siRNA or shRNA are controls for the fractionation procedure. Vimentin served as a marker for the CF and GAPDH for the SF.

To elucidate the molecular mechanisms by which LOXL2 and L2Δ13 modulate glycolysis, we focused on the glycolytic enzymes that we have identified as enzymes associated with LOXL2/L2Δ13. Overexpression of either LOXL2 or L2Δ13 did not significantly alter the total protein expression levels of aldolase A, GAPDH and enolase *in vitro* and *in vivo* (Supplementary Figs. S6A and S6B). Aldolase A maintains glucose homeostasis depending on its enzymatic activity and contributes to glycolysis [25]. Total aldolase activity was increased in nonmalignant esophageal epithelial cells expressing either LOXL2 or L2Δ13, as well as in L2Δ13-overexpressing mice (Supplementary Fig. S6C). In agreement with these observations, the catalytic activity of aldolase was reduced following depletion of LOXL2 in esophageal cancer cells (Fig. 4A; Supplementary Fig. S6D). The decrease in glycolysis due to suppressed aldolase activity was fully reversed following re-expression of LOXL2/L2Δ13, raising the possibility that LOXL2 and L2Δ13 boost glycolysis by regulating aldolase activity (Fig. 4B).

Glycolysis is organized around the actin cytoskeleton and binding of aldolase to filamentous actin inhibits its catalytic activity [[26], [27], [28]]. To determine whether LOXL2/L2Δ13 interferes with the binding of aldolase A to the cytoskeleton, cells were permeabilized with digitonin to enable access of diffusible aldolase, followed by the collection of supernatant and cell lysates for further analyses of soluble and immobilized aldolase. Both LOXL2 and L2Δ13 induced the release of aldolase A in normal esophageal epithelial cells overexpressing cDNA encoding LOXL2 or L2Δ13 (Fig. 4C). Mobilization of aldolase A was prevented following the silencing of LOXL2 expression in esophageal cancer cells, which were conversely restored by re-expression of LOXL2 or L2Δ13 in cells silenced for LOXL2 expression (Fig. 4D and E; Supplementary Fig. S6E). The observed effects of LOXL2/L2Δ13 on glycolysis acceleration display similarities with the conventional effects of the serine/threonine kinase Akt on glycolysis [29]. However, LOXL2/L2Δ13 mobilized aldolase A in an Akt-independent manner as illustrated by Akt S473 phosphorylation which remained constant regardless of the presence or absence of LOXL2 or L2Δ13 (Fig. 4C–E; Supplementary Fig. S6E). Likewise, LOXL2-or L2Δ13-induced changes in the concentration of diffusible aldolase A were accompanied with a parallel alteration in the enzymatic activity of aldolase A in the supernatant derived from both nonmalignant and malignant cells (Fig. 4F–H; Supplementary Fig. S6F). Cell fractionation indicated that both LOXL2 and L2Δ13 cause a shift of aldolase A from the cytoskeletal to the cytosolic fraction, whereas silencing LOXL2 expression inhibited the mobilization of aldolase A of esophageal cancer cells (Fig. 4I–K; Supplementary Fig. S6G). Therefore, LOXL2/L2Δ13 stimulates aldolase mobilization to enhance its enzymatic activity, which in turn promotes glycolysis and tumor cell proliferation.

### 2.5 LOXL2 and L2Δ13 deacetylate metabolic proteins including aldolase A

**Fig. 5:**
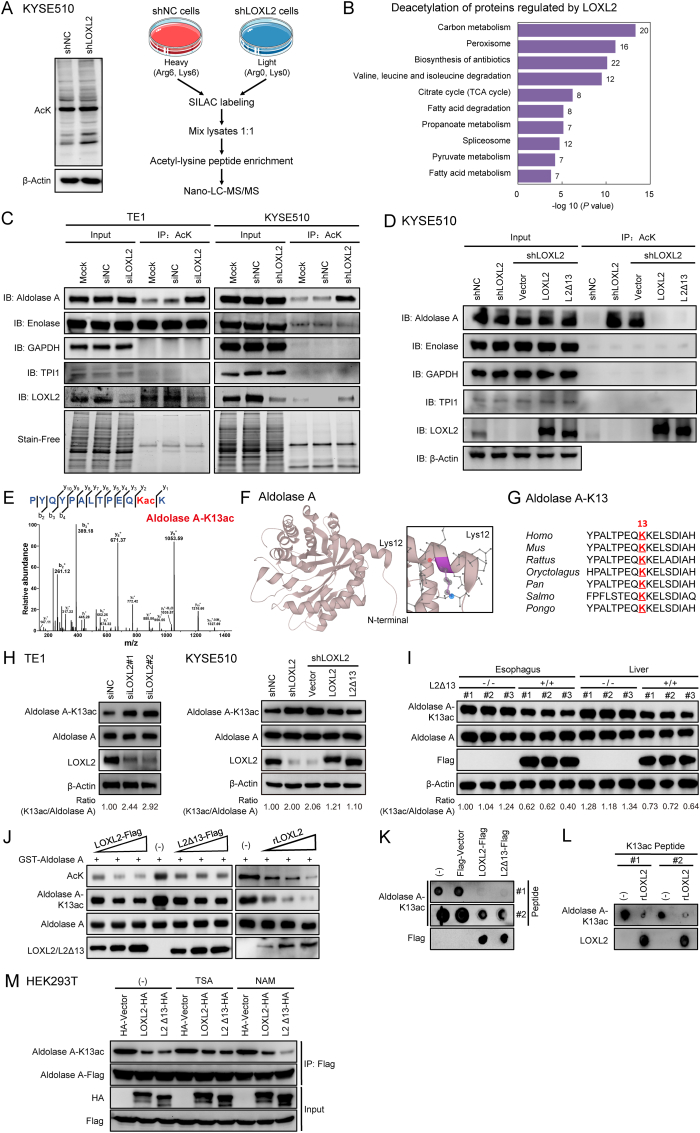
LOXL2/L2Δ13 catalyzes deacetylation of aldolase A-K13. **(A)** Left: acetylation level of total proteins from whole cell lysates of KYSE510 cells depleted for LOXL2; right: schematic diagram of the analytical strategy for SILAC labeling and global profiling of lysine acetylation. Control and LOXL2-depleted KYSE510 cells were separately labeled with “heavy” and “light” arginine and lysine using SILAC, and then proteins were digested for LC-MS/MS analysis. **(B)** Significant patterns show KEGG enrichment pathways of acetylated proteins in MS analysis. **(C)** Total proteins from untreated cells (mock), cells with scrambled siRNA or shRNA (siNC or shNC) and LOXL2-silenced cells were immunoprecipitated using acetyl-Lys and probed with indicated antibodies. Stain-Free gels was used as the control for equal protein concentration for the IP. **(D)** Total proteins from KYSE510 stably-infected cells were immunoprecipitated using acetyl-Lys and probed with indicated antibodies. Beta-Actin served as the control for equal protein concentration for the IP. **(E)** Endogenous aldolase A was purified from LOXL2-depleted and control KYSE510 cells with SILAC labeling, and acetyl peptides regulated by LOXL2 were identified. Shown are representative MS/MS spectra of acetylated aldolase A-K13. **(F)** Crystal structure model of human aldolase A (PDB database accession: 1ALD). Lys 12 in the model indicates ALDOA-K13 in our study. **(G)** Sequence alignment of acetylation sites of aldolase A-K13 from different species. **(H, I)** Aldolase A acetylated at K13 (aldolase A-K13ac) and total aldolase A expression level in indicated cells, esophagi and livers of mice. **(J)** Purified GST-aldolase A from bacteria were incubated with increasing amount of purified LOXL2-Flag/L2Δ13-Flag from HEK293T transfectants or recombinant LOXL2 protein for *in vitro* LOXL2/L2Δ13 deacetylase activity assays. **(K)** Indicated acetyl peptides of the aldolase A-K13 were incubated with the purified LOXL2-Flag, L2Δ13-Flag and Flag-vector proteins from HEK293T transfected cells. The reaction products were blotted with anti-aldolase A-K13ac or anti-Flag. **(L)** The acetyl peptides of the aldolase A-K13 were incubated with the purified recombinant LOXL2 protein. **(M**) HEK293T cells were transfected with the indicated plasmids and treated with or without traditional histone deacetylase inhibitors. Aldolase A acetylation was analyzed with an antibody against acetyl-Aldolase A (K13).

Lysyl oxidase family member LOXL3 is found to catalyze deacetylation and deacetylimination reactions, in addition to lysine amine oxidation, in cell proliferation and inflammatory responses [30]. The acetylation level of proteins in KYSE510-derived cell lysates increased upon depletion of LOXL2, and was reduced following LOXL2/L2Δ13 re-expression (Fig. 5A; Supplementary Fig. S7A). A reduction in global acetylation was also observed in L2Δ13-overexpressing mice as compared with wild-type mice (Supplementary Fig. S7B), suggestive of an extensive protein deacetylation function of LOXL2/L2Δ13. To monitor quantitative changes in LOXL2-regulated acetylation, we performed stable isotope labeling of amino acids in cell culture (SILAC) followed by affinity enrichment of lysine acetylation using nano-LC-MS/MS to systematically compare the acetyl peptide levels of KYSE510 cells before and after LOXL2 depletion (Fig. 5A). Proteomic analysis demonstrated that silencing LOXL2 expression altered the acetylation state of 249 proteins (331 sites) by greater than 1.5-fold, in which 241 (323 sites) were upregulated and only eight were downregulated (Supplementary Table S1). Proteins acetylated by LOXL2 depletion were mainly involved in metabolic processes including glucose metabolism, fatty acid metabolism and amino acid metabolism (Fig. 5B). Co-immunoprecipitation experiments were subsequently performed to validate the enhanced acetylation levels of some of the metabolic proteins that were identified in the proteomic screen. Intriguingly, only one of the LOXL2/L2Δ13-interacting proteins, aldolase A, was hyperacetylated in LOXL2-silenced esophageal cancer cells (Fig. 5C), while re-expression of LOXL2 or L2Δ13 reduced the elevated acetylation level of aldolase A (Fig. 5D). These results strongly suggest that LOXL2 and L2Δ13 induce aldolase deacetylation due to their physical interaction with aldolase A.

To identify the lysine residues of aldolase A that are deacetylated by LOXL2/L2Δ13, we purified SILAC- labeled endogenous aldolase A from LOXL2-depleted and control KYSE510 cells. The K13 residue of aldolase A was hyperacetylated in LOXL2-depleted cells but not in control cells (Fig. 5E; Supplementary Table S1). According to sequence alignment analysis, the K13 residue (K12 in the PDB-1ALD structure model) is highly conserved between diverse animal species (Fig. 5F and G). We further synthesized acetyl peptides derived from the aldolase A-K13 and generated a polyclonal rabbit antibody specifically directed against the acetylated K13 residue of human aldolase A (Supplementary Fig. S7C). Aldolase A-K13 was frequently acetylated in nonmalignant cells and different types of esophageal cancer cells (Supplementary Fig. S7D). Interestingly, depletion of LOXL2 prominently enhanced the acetylation of aldolase-K13 (Fig. 5H; Supplementary Fig. S7E). Overexpression of LOXL2 or L2Δ13 conversely inhibited aldolase A-K13 acetylation in both cancer cells and mice (Fig. 5H and I; Supplementary Fig. S7E). To directly measure the aldolase A-targeted deacetylase activity of LOXL2 and L2Δ13, we performed an *in vitro* aldolase deacetylation assay using purified Flag-tagged LOXL2, Flag-tagged L2Δ13 or recombinant LOXL2 and GST-tagged aldolase A proteins. Both LOXL2 and L2Δ13 efficiently catalyzed aldolase A deacetylation at the K13 locus in a concentration-dependent manner (Fig. 5J; Supplementary Fig. S7F). In agreement with these observations, two different K13 acetyl peptides were also specifically deacetylated by LOXL2 and L2Δ13 *in vitro* (Fig. 5K and L). Neither trichostatin A (TSA) nor nicotinamide (NAM) was able to block the LOXL2/L2Δ13-directed deacetylation of aldolase A-K13 (Fig. 5M; Supplementary Fig. S7G). These results identify aldolase A as a LOXL2 deacetylation substrate and demonstrate that this activity of LOXL2 is independent from its conventional lysyl-oxidase activity.

### 2.6 LOXL2/L2Δ13-dependent deacetylation of aldolase A-K13 induces metabolic reprogramming in tumor progression

**Fig. 6:**
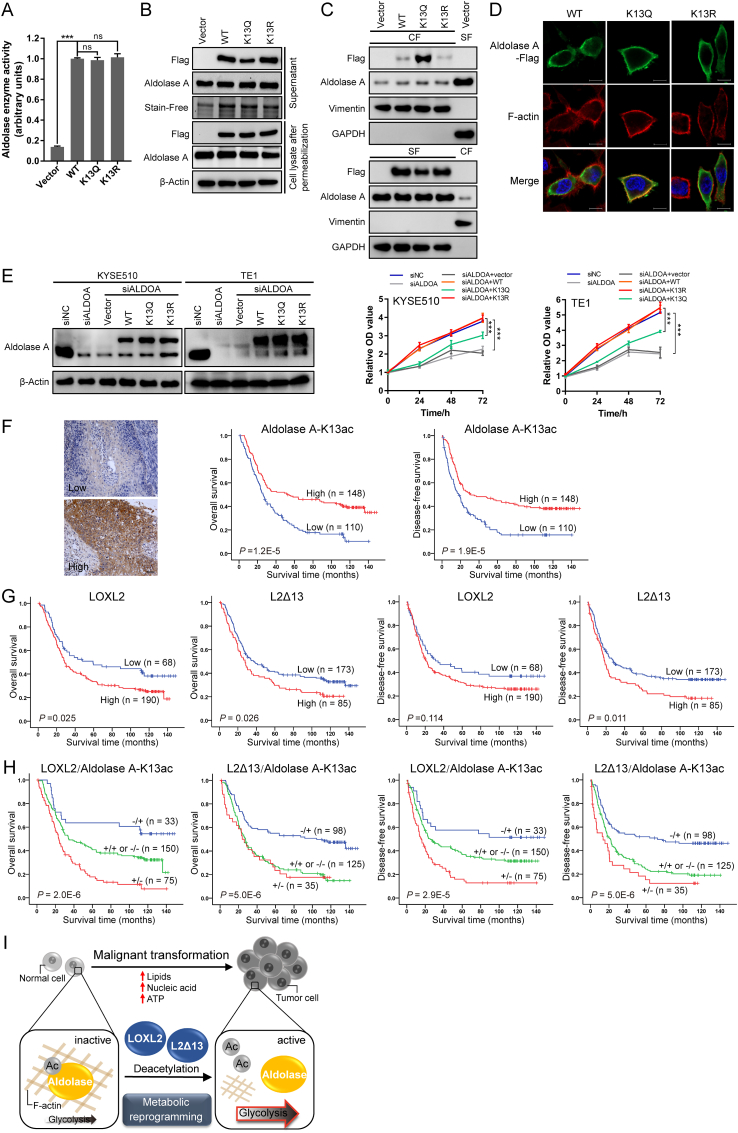
LOXL2-dependent deacetylation of aldolase A-K13 induces metabolic reprogramming in esophageal cancer progression. **(A)** Flag-tagged wild-type aldolase A, acetyl-mimetic mutant aldolase A-K13Q and non-acetylatable mutant aldolase A-K13R were expressed in KYSE510 cells. Aldolase A proteins were purified by IP with Flag antibody, and aldolase activity was determined. Cells transfected with Flag-tagged vector served as the negative control. Data represent mean ± SD (n = 3). ***P < 0.001; ns, not significant. **(B)** Western blotting of supernatant and cell lysate of KYSE510 cells transfected with empty vector, Flag-tagged aldolase A (WT), its acetyl-mimetic or non-acetylatable mutant, including K13Q and K13R. **(C)** KYSE510 cells expressing Flag-tagged wild-type aldolase A, its acetyl-mimetic mutant K13Q and non-acetylatable mutant K13R were lysed and fractionated. Vimentin is used as a marker for the CF and GAPDH for the SF. Fractions from the cells transfected with Flag-tagged empty vector are controls for the fractionation procedure. **(D)** Immunofluorescence analysis of the effect of different aldolase A mutants from **(B)** on its release from the actin cytoskeleton in KYSE510 cells. Scale bar, 10 μm. **(E)** Left: Western blotting of aldolase A in the control cells, cells silenced for aldolase A expression, cells transfected with empty vector, Flag-tagged aldolase A (WT), its K13Q and K13R mutants; right: MTS assays of the indicated cells. **(F)** Representative low and high acetylation of aldolase A-K13 by IHC analysis (left) and Kaplan-Meier curves by log-rank test (right) of aldolase A-K13ac in tissue microarrays of patients with esophageal cancer (n = 258). Scale bar, 50 mm. **(G)** Kaplan-Meier curves by log-rank test of LOXL2 and L2Δ13 for overall survival and disease-free survival in patients of **(F)**. **(H)** Kaplan-Meier estimates of overall survival and disease-free survival of patients with esophageal cancer according to LOXL2 or L2Δ13 associated with acetylated aldolase A-K13. **(I)** Proposed model integrating the LOXL2/L2Δ13-activated metabolic reprogramming through deacetylating glycolytic proteins in tumorigenesis and tumor progression.

The acetylation levels of glycolytic enzymes correlate with their enzymatic activities in primed human pluripotent stem cells [31]. Neither the acetyl-mimetic mutant of aldolase, K13Q, nor its non-acetylatable mutant, K13R, had any direct effect on the aldolase enzymatic activity in esophageal cancer cells (Fig. 6A). However, the K13Q acetyl-mimetic mutant, but not the K13R non-acetylatable mutant, significantly prevented the release of aldolase A in the cytoplasm (Fig. 6B). Cell fractionation experiments confirmed that aldolase acetylation at K13 impeded the dissociation of aldolase A from the cytoskeletal compartment, which was further verified by colocalization analysis of aldolase A and actin fibers (Fig. 6C and D). These observations suggest that deacetylation of aldolase A by LOXL2 and L2Δ13 promotes the mobilization of aldolase from the actin cytoskeleton, thus aldolase A-K13 deacetylation can regulate glycolysis.

To elucidate the effects of acetylation of aldolase A-K13 on tumor progression, we conducted loss- and gain-of function assays in esophageal cancer cells. Compared to the control KYSE510 and TE1 cells, silencing aldolase A expression greatly inhibited cancer cell proliferation (Fig. 6E). By contrast, overexpression of wild-type aldolase A and its K13R mutant stimulated the proliferation of esophageal cancer cells, but these effects were weaker in cells that expressing aldolase A-K13Q (Fig. 6E). The contribution of the acetylation status of aldolase-K13 to glycolysis in esophageal cancer cells prompted us to determine its prognostic value in clinical settings. We therefore measured the levels of K13 acetylated aldolase A by immunohistochemical staining of tumor sections derived from 258 esophageal cancer patients. Patients displaying high level acetylation of aldolase A had longer overall survival and disease-free survival as compared with patients in which aldolase A was less acetylated (Fig. 6F). Moreover, hyperacetylated aldolase A correlated with histologic grade and tumor invasion state and was found to represent an independent prognostic factor for favorable clinical outcome (Supplementary Tables S2 and S3). Consistently with our previous study [16], elevated LOXL2 or L2Δ13 was significantly associated with poor survival in these patients (Fig. 6G). We subsequently combined the states of either LOXL2 or L2Δ13 and K13-acetylated aldolase A to develop molecular models for predicting the clinical prognosis of patients with esophageal cancer. Indeed, low level expression of LOXL2 or L2Δ13 was associated with increased acetylation of aldolase A-K13, and was significantly correlated with longer overall survival and disease-free survival (Fig. 6H).

To conclude, our experiments identify LOXL2 as a novel deacetylase that directly interacts and deacetylates aldolase A. This in turn induces its release from filamentous actin and enzymatic activity, leading to increased glycolysis which subsequently contributes to tumor development and tumor metastasis (Fig. 6I).

## 3 Discussion

Previous studies have shown that the pro-fibrogenic effects of LOXL2 are dependent on its extracellular amine oxidase activity. On the other hand, the pro-tumorigenic effects of LOXL2 are mediated by a combination of extracellular and intracellular activities of LOXL2 [3,4,[7], [8], [9], [10]]. Here we found that LOXL2 and its non-secreted catalytically inactive L2Δ13 splice isoform are able to regulate metabolic reprogramming so as to promote tumor initiation and progression *in vitro* and *in vivo*. We further showed that both L2Δ13 and LOXL2 bind to several glycolysis-promoting enzymes, including aldolase A. In the case of aldolase A, this interaction triggered the deacetylation of aldolase on the K13 residue. This in turn led to enhanced glycolysis that ultimately contributes to tumor growth and tumor metastasis in esophageal cancer.

Tumor cells preferentially uptake, transfer and utilize glucose at much higher rates in order to generate ATP, maintain redox balance and support biosynthesis, thereby reprogramming their metabolism and that of other cells in the tumor microenvironment, as compared with the metabolism of normal cells [32]. Loss- and gain-of-function assays showed that, in addition to the effects of LOXL2 on tumor cell invasion and tumor metastasis [16,[33], [34], [35]], both L2Δ13 and LOXL2 promote esophageal cancer cell proliferation and tumor growth in xenograft experiments. The Warburg effect is characterized by enhanced glycolysis and lactate production regardless of oxygen availability, and represents a hallmark of cancer cells [24,36]. Interestingly, we found that a similar enhancement of glycolysis takes place in normal esophageal epithelial cells following overexpression of either LOXL2 or L2Δ13. In contrast, depletion of LOXL2 in esophageal cancer cells inhibits glycolysis, suggesting that glycolysis induced by L2Δ13/LOXL2 contributes to the Warburg effect and tumor progression.

We identified and validated aldolase A, GAPDH, and alpha-enolase as glycolysis-associated proteins that physically interact with LOXL2 and L2Δ13. Aldolase A catalyzes the conversion of fructose-1,6-bisphosphate (FBP) to glyceraldehyde-3-phosphate (GA3P) and dihydroxyacetone phosphate (DHAP), providing a major source of substrates required for nucleotide and purine biosynthesis [25]. Our findings suggest that both the increased glycolysis and cell proliferation observed in esophageal cancer is due, at least in part, to the interactions of LOXL2/L2Δ13 with aldolase A. Aberrant expression of aldolase A is found to be associated with tumor metastasis and worse patient prognosis in several types of solid tumors, including lung cancer and pancreatic ductal adenocarcinoma [37,38]. Inhibition of aldolase A is sufficient to block glycolysis, thereby inhibiting the uncontrolled cell proliferation of tumor cells under hypoxic conditions [39]. We found that silencing L2Δ13/LOXL2 expression inhibits the enzymatic activity of aldolase A without affecting its expression. Aldolase A binds to and accumulates on actin fibers through electrostatic bonds associated with its FBP substrate [[26], [27], [28]]. Our results suggest that L2Δ13 and LOXL2 control the release of aldolase from filamentous actin and simultaneously stimulate the enzymatic activity of aldolase A via its deacetylation on K13. It is reported that aldolase A catalyzes the conversion of FBP into GA3P and DHAP in an energetically unfavorable multistep reaction, which is slower than the preceding phosphorylation and subsequent oxidation steps of glycolysis [40]. Moreover, aldolase A has a low turnover rate (k_cat_) as compared with the upstream hexokinase or phosphofructokinase [40,41]. The abundance of active aldolase A in the cytoplasm probably becomes rate limiting to glycolytic flux. Thus, LOXL2-induced aldolase A activation via recruitment from the cytoskeleton is a rapid and efficient way for cancer cells to increase metabolic flux. We have previously demonstrated that cytoplasmically localized LOXL2, as well as L2Δ13, induces cytoskeletal reorganization to promote tumor invasion and metastasis [16]. This LOXL2/L2Δ13-induced reorganization may also trigger aldolase release into the cytoplasm to further enhance tumor progression. Taken together, our results demonstrate that the lysyl-oxidase-independent effects of L2Δ13/LOXL2 contribute to tumor progression via enzyme mobilization and enhanced glycolysis.

Evolutionarily conserved lysine acetylation regulates the stability, activity, and localization of metabolic enzymes in response to extracellular changes [42]. Histone deacetylase (HDAC) and Sirtuin (SIRT) are two classical families responsible for the removal of acetyl groups from lysine side chains of acetylated proteins, including some glycolytic enzymes [43]. Moreover, LOXL3 of the lysyl oxidase family was previously reported to be a specific STAT3 deacetylase with N-terminal scavenger receptor cysteine-rich (SRCR) repeats 1–3 bearing the major deacetylation activity [30]. Our results concerning the deacetylase activity of LOXL2 are in agreement with this prior study on the contributions of the deacetylase activity of LOXL3 to inflammatory responses [30]. Surprisingly, the amino acid sequences of LOXL2 and L2Δ13 are extremely different from multiple classical deacetylases (including HDAC1-9 and SIRT1-7) with the similarity rate of only 3.29–11.35%. However, LOXL2 and L2Δ13 share more than 50% of the same amino acid sequences as LOXL3, and their C-terminal domain and N-terminal SRCR repeats domain are also highly homologous with high ββ sheet contents (Supplementary Fig. S8). Therefore, we speculate that the LOXL2-mediated protein deacetylation reaction is similar with that by LOXL3.

Full-length LOXL2 is a dual-specificity enzyme involved in deamination and/or deacetylation reactions, whereas L2Δ13 completely lacks the amine oxidase activity of LOXL2 and contains 18 unique amino acids at the C-terminus [20]. Here, our *in vitro* and *in vivo* results showed that L2Δ13 facilitates glycolysis and tumor cell proliferation more efficiently than full-length LOXL2 in tumor progression. Mechanistically, L2Δ13 was sufficient to specifically catalyze the deacetylation of aldolase A on the K13 residue and even stimulated aldolase activity more strongly than full-length LOXL2 during malignant transformation. In clinical settings, high expression level of L2Δ13 combined with hypoacetylated aldolase A-K13 predict a higher mortality risk in patients with esophageal cancer. These data clearly demonstrate that the proliferation- and metabolism-promoting effects of LOXL2 in cancer are mainly directly linked to its deacetylase activity, but not its classical amine oxidase activity.

L2Δ13/LOXL2 deacetylates aldolase on K13 and facilitates its release from the filamentous actin, which may be due to the location of K13 in a positively-charged surface region of aldolase that mediates its attachment to actin [27]. More importantly, we extended these results to identify a subgroup of esophageal cancer that exhibit LOXL2 or L2Δ13 combined with decreased acetylation of aldolase-K13. These findings identify LOXL2 as a promising metabolic target for cancer therapy, particularly in personalized therapy of patients with esophageal cancer.

To summarize, we provide compelling evidence that identifies LOXL2 and L2Δ13 as novel deacetylates with physiological and pro-tumorigenic functions in cell metabolism. Our findings highlight a hitherto-unknown mechanism by which LOXL2 and L2Δ13 catalyze the deacetylation of aldolase to stimulate its mobilization and enzymatic activity in glycolysis that in turn promotes tumor progression.

## 4 Materials and methods

### 4.1 Patients and tissue specimens

Human esophageal squamous cell carcinoma tissue specimens were collected directly after surgical resection between November 2007 and January 2011 at Shantou Central Hospital (Sun Yat-sen University, Shantou, China). The study was approved by institutional review board, and written informed consent was obtained from all patients.

### 4.2 Xenograft assays

Xenograft assays in nude mice were performed as described previously [44]. KYSE510 cells infected with scrambled shRNA (shNC) or shLOXL2 lentivirus (shLOXL2), or stable LOXL2-silenced KYSE510 cells expressing empty vector (vector), LOXL2-Flag (LOXL2) or L2Δ13-Flag (1 × 10^6^ cells in 100 μL PBS) were injected subcutaneously into the left flanks of BALB/c nude mice (Vital River Laboratories) at the age of 5 weeks (n = 7 per group). General mouse behaviors were monitored and the tumor volume was measured every 3 days. The tumor size was calculated by the formula volume = 1/2 length × width [2]. Mice were euthanized after 30 days and tumor tissues were excised for growth analysis.

### 4.3 ATP and glucose uptake assays

Cells were seeded into 6-well plates and were transfected or infected with the indicated constructs. After 48 h, cells were harvested and 1 × 10^4^ cells were subsequently plated into a 96-well plate and cultured for 8 h. Cells were collected, extracted and incubated using the CellTiter-Glo® Luminescent Cell Viability Assay (G7570, Promega) for ATP level analysis. Moreover, the Glucose Uptake-Glo™ Assay (J1342, Promega) was performed to measure glucose uptake in cells based on the detection of 2-deoxyglucose-6-phosphate (2DG6P). Luminescence was measured and recorded on a GloMax® luminometer (Promega). The group of wells containing medium without cells served as negative controls.

### 4.4 Lactate production assays

For lactate production assays, cells were transfected or infected as for the ATP quantitation. After the determination of cell number, 5 × 10^4^ cells were plated into a 24-well plate and cultured for 12 h. To measure the secretion of lactate in the cell supernatants, the medium was collected and deproteinized with a 10 kDa MWCO spin filter to remove lactate dehydrogenase. After adding 200 μL Lactate Assay Buffer of the Lactate Assay Kit (MAK064, Sigma-Aldrich) to each sample, cell lysate was centrifuged at 13,000×*g* for 10 min to remove insoluble material and then was deproteinized as described for the medium. Subsequently, 2.5 μL of prepared medium of cells and 10 μL of prepared cell lysis were brought to a final volume of 50 μL/well with Lactate Assay Buffer. To measure lactate production, each of the reaction content per well was incubated with 50 μL of Master Reaction Mix for 30 min at room temperature, protected from light. The reaction mixture was measured at 570 nm based on colorimetric assays.

### 4.5 Cell permeabilization and fractionation

For aldolase activity in the diffusible fraction (in the supernatant), cells were seeded into 6-well plates and permeabilized with digitonin (30 μg/mL; Sigma-Aldrich), followed by the collection of supernatant and cell lysate for further analyses as described previously [28]. For cell fractionation, ProteoExtract®Cytoskeleton Enrichment and Isolation Kit (Millipore) was applied to isolation of cytoskeletal and soluble compartments of indicated cells.

### 4.6 Pull-down assays

KYSE510 cells or LOXL2-Flag/L2Δ13-Flag transfected HEK293T cells were harvested in bicine buffer (Thermo scientific). GST-Aldolase A fusion proteins were immobilized on glutathione resins, and then incubated with the cell lysates or purified recombinant Flag-tagged LOXL2/L2Δ13 at 4 °C for 2 h. Following precipitation, the cell pellets were washed 3 times with the lysis buffer (pH 7.3) containing 4.3 mM Na_2_HPO_4_, 1.47 mM KH_2_PO_4_, 137 mM NaCl and 0.1% Triton X-100, and ultimately analyzed by Western blotting with indicated antibodies.

### 4.7 *In situ* proximity ligation assay (PLA)

Duolink *in situ* PLA (DUO92014, Sigma-Aldrich) was performed in esophageal cancer cells. The paired-primary antibodies used in the present study were either rabbit anti-LOXL2, L2Δ13 or GAPDH antibody with mouse anti-aldolase A antibody. As a negative control, PLA was performed using only anti-LOXL2, anti-L2Δ13 or anti-aldolase A antibody, respectively. Briefly, cells were fixed with 4% paraformaldehyde for 10 min, washed three times with PBS, and permeabilized in 0.1% Triton X-100 for 10 min, followed by incubation with the indicated antibody pairs overnight at 4 °C. PLA was performed according to the manufacturer's recommendations. PLA imaging was conducted on a laser-scanning confocal microscope (LSM 800, Carl Zeiss) with a 63 × , 1.40 NA, Plan-apochromatic oil objective.

### 4.8 *In vitro* deacetylation assay

Purified GST-tagged aldolase A (0.5 μg) was incubated with purified increasing amount (0.25 μg, 0.5 μg and 1 μg) of Flag-tagged LOXL2/L2Δ13 or recombinant human LOXL2 (>95% purity; Sino Biological Inc.) in a 50 nM ATP-containing reaction buffer at 30 °C for 2 h. The buffer contains 50 mM Tris-HCl (pH 9.0), 50 mM NaCl, 4 mM MgCl_2_, 0.5 mM DTT, 0.2 mM phenylmethylsulfonyl fluoride, 0.02% Nonidet P-40 and 5% glycerol [45]. Under the same condition, 0.5 μg synthetic acetyl peptide of aldolase A-K13 (98% purity; PTM Biolabs Inc.) was incubated with 0.25 μg purified Flag-tagged LOXL2/L2Δ13 or recombinant human LOXL2. The 100 μL total reaction mixtures were stopped with 5 × SDS loading buffer, and 20 μL or 2 μL reaction products were subjected to Western blotting or Dot blotting analysis, respectively.

### 4.9 Metabolomics

Liver tissues were harvested from wild-type (n = 12) and L2Δ13-overexpressing mice (n = 12), rinsed with saline solution and immediately transferred to a tube placed in liquid nitrogen. The frozen tissues were minced, weighed, and 25 mg was dissolved in an equal volume of water:methanol (1:1, v:v) solution. Samples were disrupted with a TissueLyser through high-speed shaking, precipitated overnight and centrifuged.

Chromatographic separations of resulting supernatant of samples were performed using an ultra-performance liquid chromatography (UPLC) system (Waters). An ACQUITY UPLC HSS T3 column (100 mm × 2.1 mm, 1.8 μm, Waters,UK) was used for the reversed phase separation. The column oven was maintained at 50 °C. The flow rate was 0.4 ml/min and the mobile phase consisted of solvent A (water + 0.1% formic acid) and solvent B (acetonitrile + 0.1% formic acid). The injection volume for each sample was 10 μL. A high-resolution tandem mass spectrometer Xevo G2 XS QTOF (Waters) was subsequently applied to detect metabolites of eluted hepatic samples in the positive and negative ion modes. Mass spectrometry data were acquired in the Centroid MSE mode and the TOF mass range was from 50 to 1200 Da (scan time, 0.2s). Differential metabolites analyzed using MetaX with a fold change ≥1.2 and *q*-value < 0.05 were considered as significant. The metabolite profile was analyzed by the Pathway Analysis module of the MetaboAnalyst.

### 4.10 Statistical analyses

Statistical analyses were performed using GraphPad Prism 7 software (GraphPad) or SPSS 22.0 software (IBM). Unless otherwise stated, all experiments were repeated independently three times. Comparative data were evaluated by an unpaired Student's t-test or nonparametric test. Survival curves were estimated using the Kaplan-Meier method with log-rank test. Differences were considered statistically significant when the two-tailed *p*-value was <0.05.

## Author contributions

J.-W. Jiao and X.-H. Zhan designed and performed the majority of the experiments, analyzed and interpreted the data, and wrote the manuscript. J.J Wang and L.-X He performed some mouse experiments and pathology analysis. Z.-C. Guo performed proteomic analysis of cancer cells. X.-E. Xu performed H&E and immunohistochemistry experiments. L.-D Liao and B. Wen assisted with cell culture and immunofluorescence. X. Huang and Y.-W. Xu performed blood chemistry analysis. H. Hu and Z.-J. Chang provided critical advice and consultations. G. Neufeld revised the manuscript and provided expertise on oncology. K. Zhang, L.-Y. Xu and E.-M. Li designed the study, provided advice and reagents, and directed the study.

## Declaration of competing interest

The authors declare no competing interests.

## Data Availability

Data will be made available on request.
