# Supplementary material for: LOXL2-dependent deacetylation of aldolase A induces metabolic reprogramming and tumor progression
Source: Redox Biol. 2022 Oct 3;57:102496. doi: 10.1016/j.redox.2022.102496 (PMC9547286; doi:10.1016/j.redox.2022.102496)
Supplement: Multimedia component 2 [file mmc2.pdf]

Figure S1

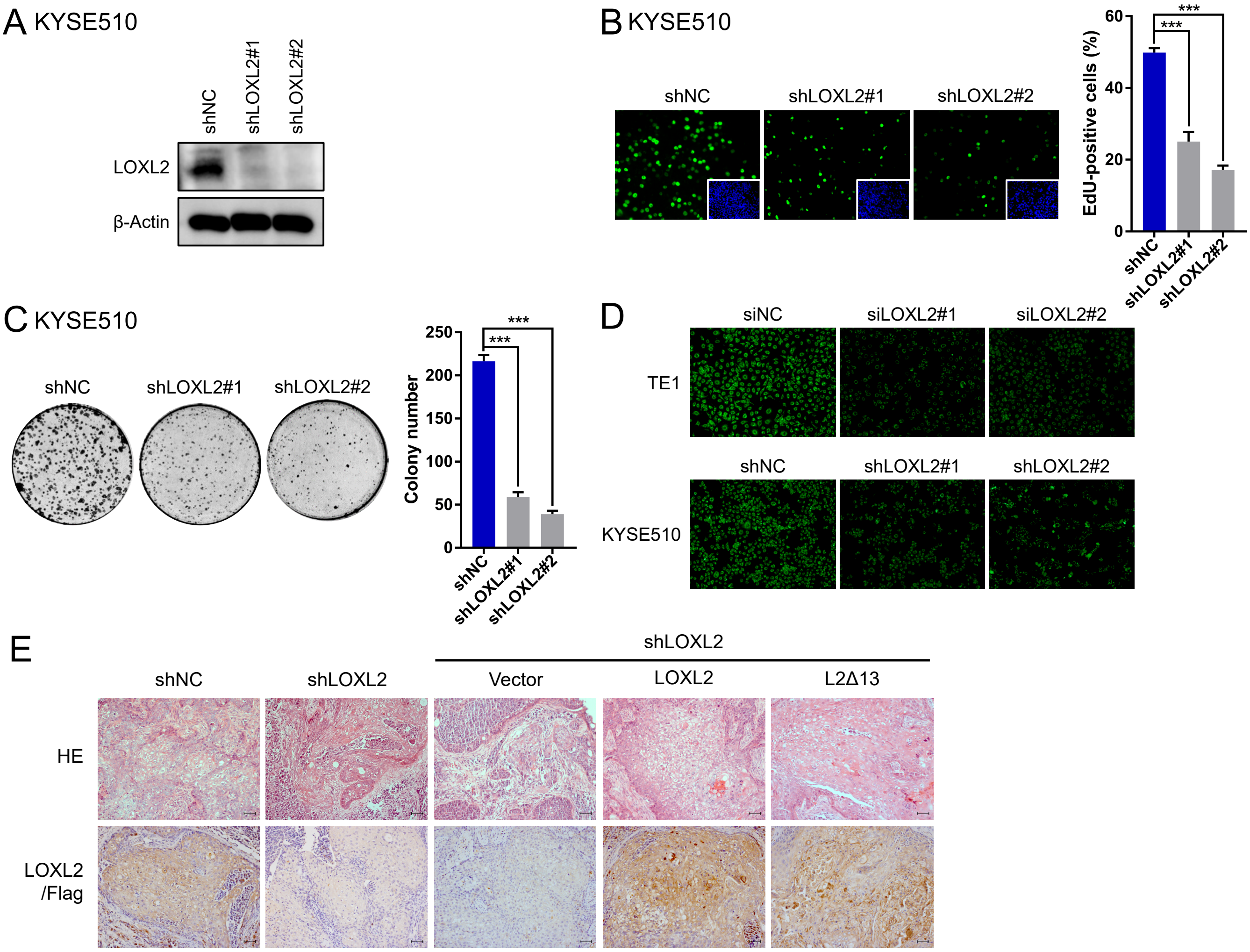

Figure S1. L2Δ13 and LOXL2 promote tumor cell proliferation *in vitro* and *in vivo*.

(A-C) Western blotting **(A)**, EdU **(B)** and colony formation **(C)** assays with esophageal cancer KYSE510 cells following LOXL2 knockdown with a specific shRNA target. Error bars indicate mean ± SD of three replicates. ***$P < 0.001$ by *t*-test analysis. **(D)** Effects of LOXL2 silencing on lipid droplets in TE1 and KYSE510 cells. **(E)** Representative paraffin sections stained with hematoxylin and eosin (H&E, top) and antibodies against LOXL2 or Flag (bottom) for tumors derived from the xenografts. LOXL2 antibody was used for the section from xenografts implanted with KYSE510 cells expressing either a scrambled (shNC) or LOXL2-silencing lentiviral vector (shLOXL2), while the Flag antibody was adopted for the remaining three groups. Scale bar, 50 μm.

Figure S2

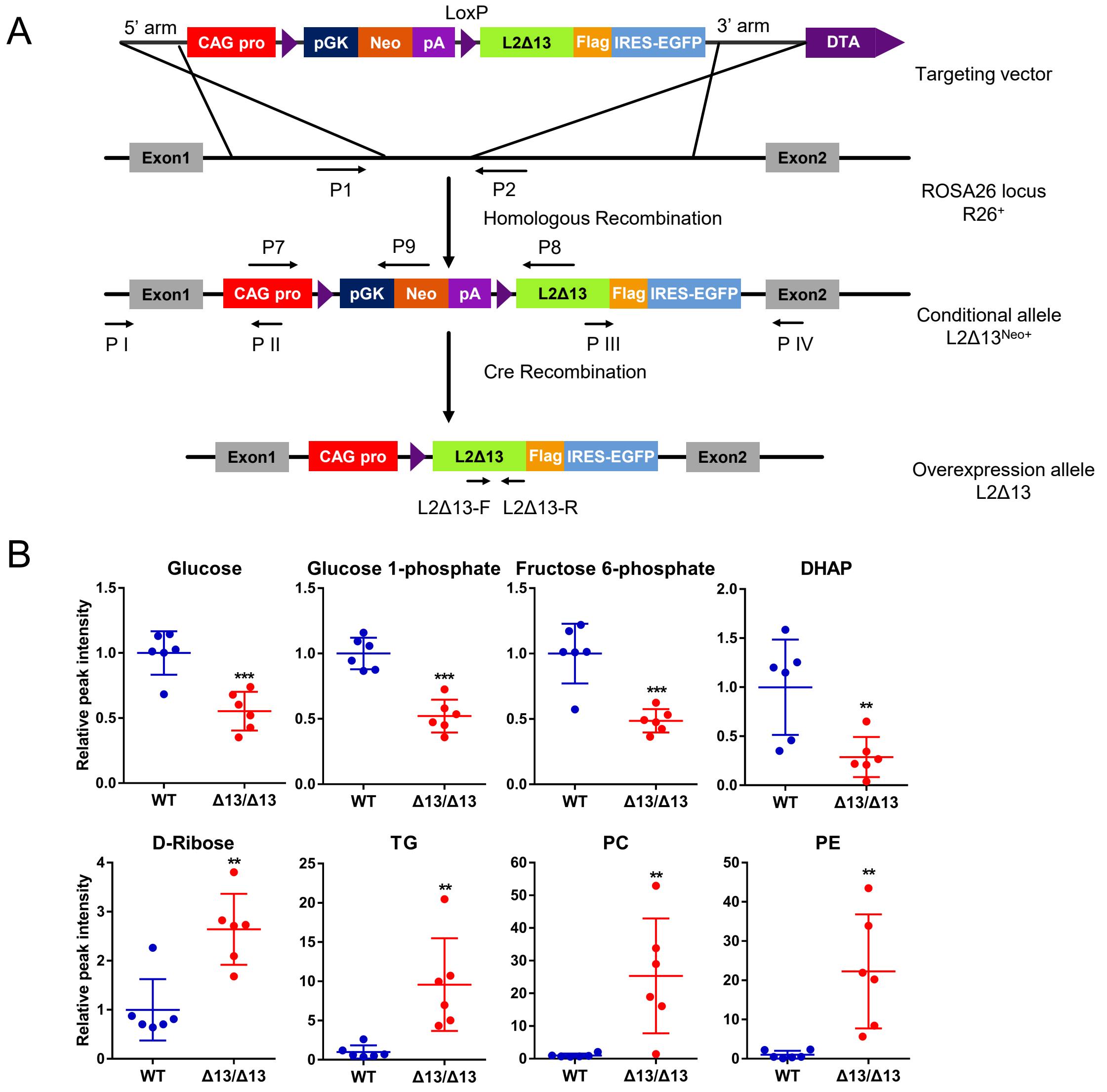

Figure S2. Generation and comparison of L2Δ13-overexpressing transgenic mice and control wild-type mice.

(A) Schematic diagram illustrating different *ROSA26* allele variants used in the study. The targeting vector contains a *PGK-neomycin-polyA* element flanked by *LoxP* sites, and the human *L2Δ13* cDNA sequence fuses to the 3X Flag sequence and the *IRES-EGFP* reporter gene. An $L2\Delta13^{Neo+}$ allele is initially generated by homologous recombination. The *PGK-neomycin-polyA* is removed upon Cre-mediated excision, then the *L2Δ13/Flag* is expressed under the control of the CAG promoter (*L2Δ13* allele). Primer pairs for PCR analysis are listed in *Supplementary Table S4*. **(B)** Representation of the downregulated glycolysis/gluconeogenesis pathways (top) and the upregulated pentose phosphate and fatty acid metabolism pathways (bottom) and relative MS peak intensity of their corresponding intermediate metabolites. $**P < 0.01$; $***P < 0.001$. *P*-value was calculated using a *t*-test. DHAP, dihydroxyacetone phosphate; TG, triglyceride; PC, phosphatidyl choline; PE phosphatidyl ethanolamine.

Figure S3

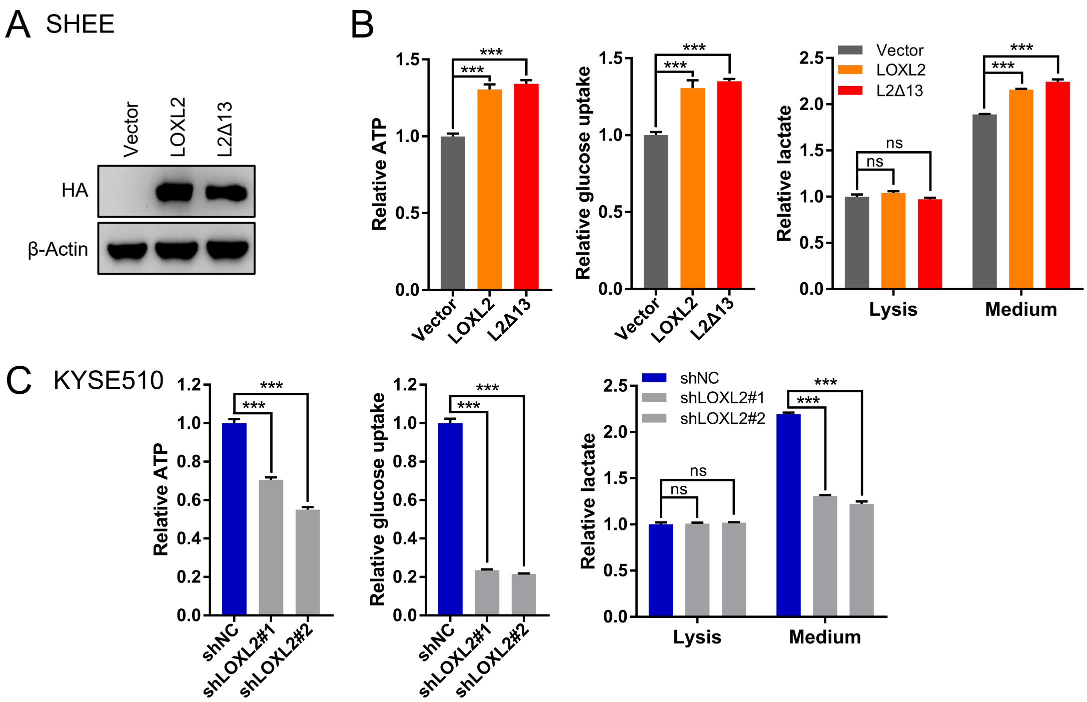

Figure S3. LOXL2 and L2Δ13 enhance glycolysis.

(A) Western blotting assays of nonmalignant esophageal epithelial cells expressing the empty vector, full-length LOXL2 or L2Δ13 variant. **(B and C)** Levels of ATP, glucose uptake and lactate of nonmalignant esophageal epithelial cells expressing recombinant full-length LOXL2, L2Δ13 variant or control vector **(B)** and esophageal cancer cells following LOXL2 silencing by specific shRNA **(C)**. Data show the mean ± SD (n = 3 or 4). ****P* < 0.001 by the *t*-test. ns, not significant.

Figure S4

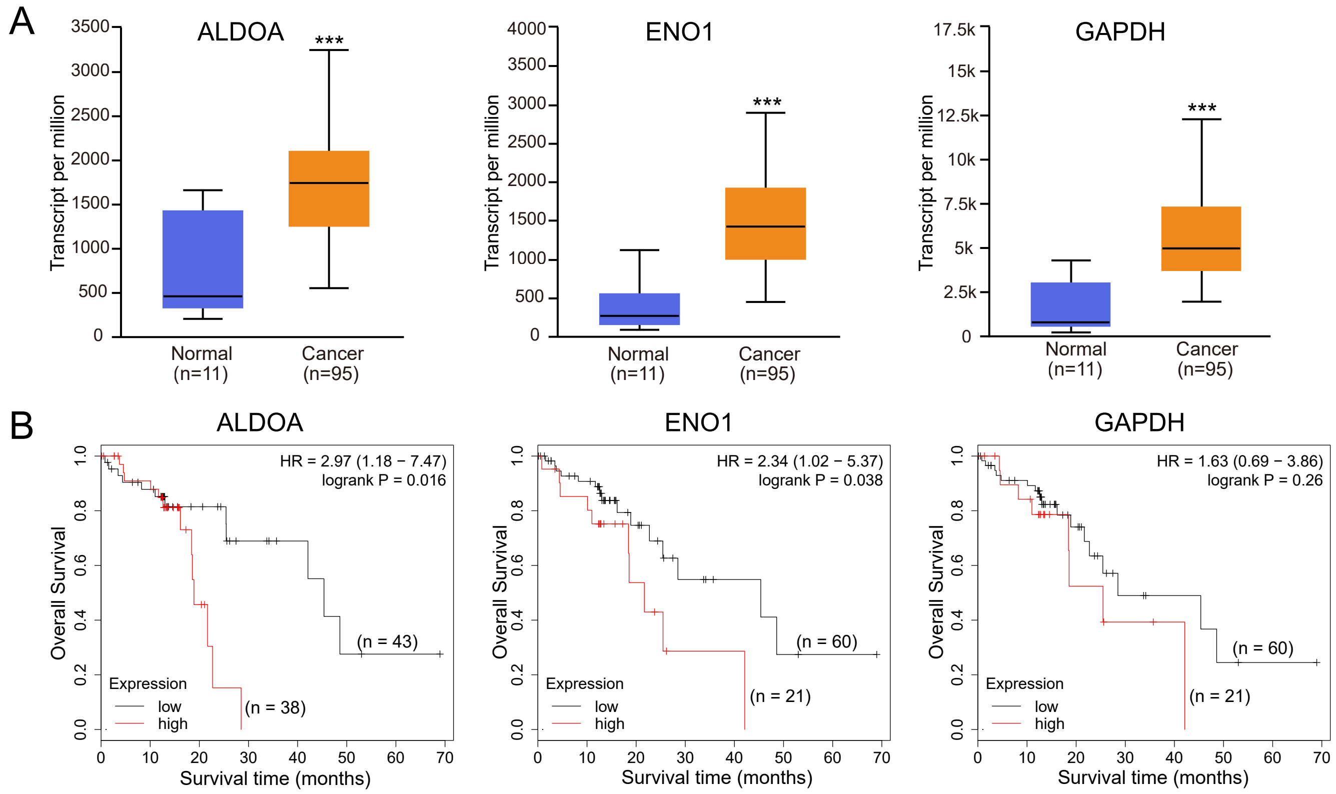

Figure S4. ALDOA, ENO1 and GAPDH predict poor clinical outcome in esophageal cancer patients.

(A) RNA-sequencing data of gene expressions of ALDOA, ENO1 and GAPDH from TCGA database in esophageal cancer (n = 95) and normal esophagus (n = 11). ****P* < 0.001 by the *t*-test. **(B)** Kaplan-Meier estimates overall survival of patients with esophageal cancer according to curves of ALDOA, ENO1 and GAPDH (n = 81; Kaplan-Meier Plotter database, http://kmplot.com/analysis/). High expression levels of these glycolic genes were associated with shorter medium survival time in patients with esophageal cancer. Statistical significance was assessed by log-rank test. HR, hazard ratio (95% CI).

Figure S5

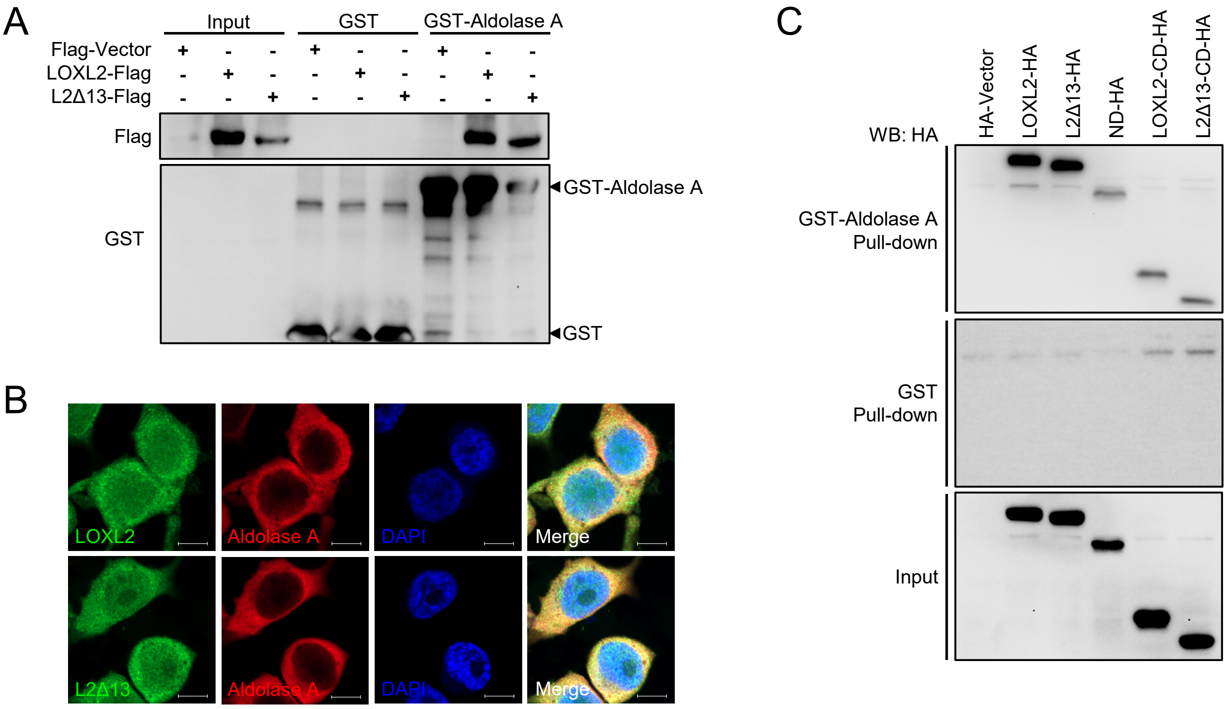

Figure S5. LOXL2 and L2Δ13 directly interact with aldolase A.

(A) GST-aldolase A and GST were retained on glutathione resins, incubated with whole cell lysates extracted from HEK293T cells transfected with LOXL2-Flag, L2Δ13-Flag or Flag empty vector, and then subjected to western blotting as indicated. **(B)** Confocal immunofluorescence staining indicate co-localization of either LOXL2 or L2Δ13 with aldolase A in KYSE510 cells that express these proteins endogenously. Shown are individual views of LOXL2/L2Δ13 (green), aldolase A (red) and nucleus (blue), and merged images showing triple staining. Scale bar, 10 μm. **(C)** Pull-down assay in which either GST-tagged aldolase A or control GST was used to pull down different HA-tagged deletion mutants of full-length LOXL2 and L2Δ13 in whole cell lysate from HEK293T cells expressing each of these mutants

Figure S6

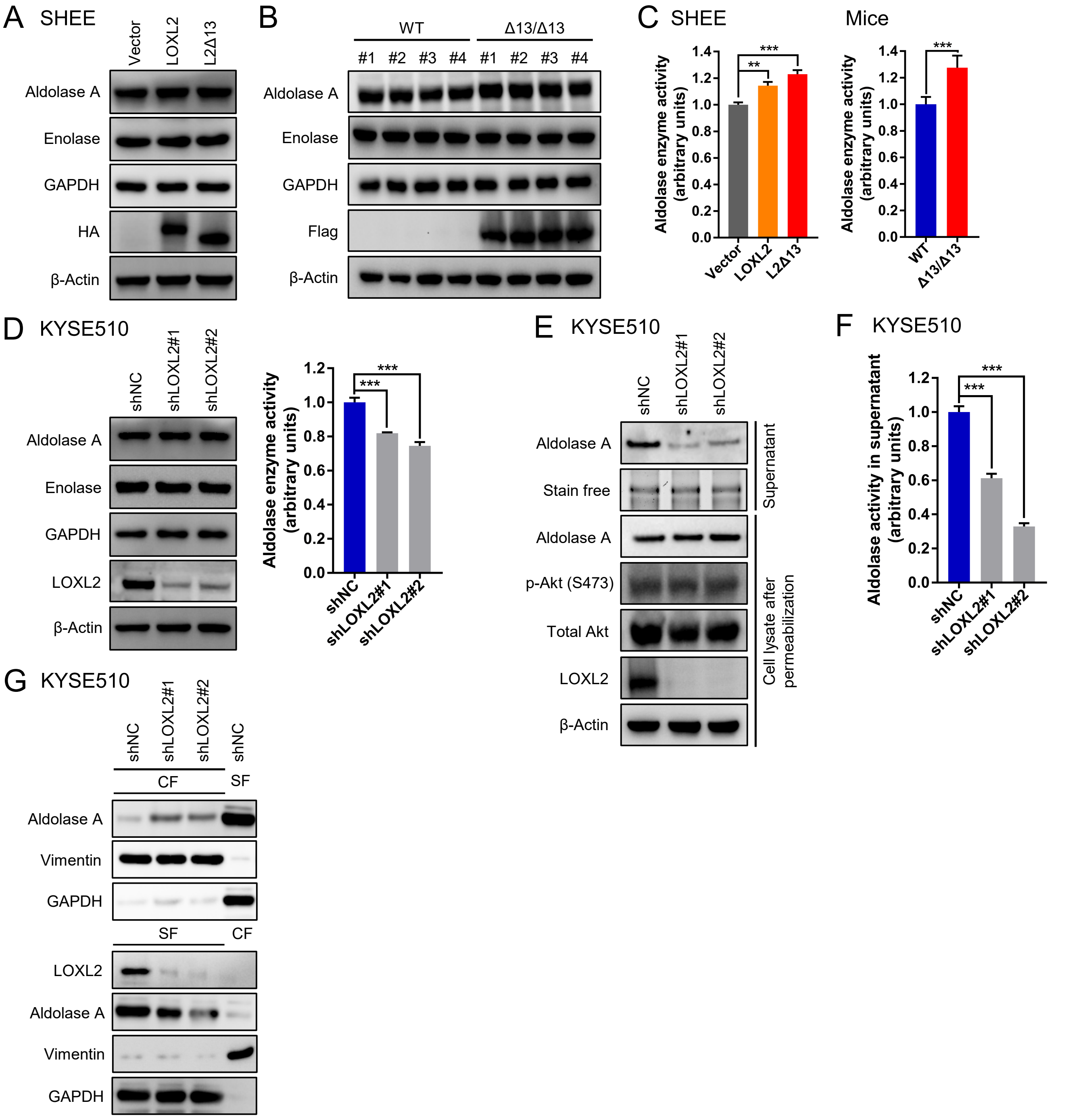

Figure S6. Depletion of LOXL2 inhibits the mobilization and enzymatic activity of aldolase A.

(A) Western blotting analysis of nonmalignant esophageal epithelial SHEE cells expressing HA-tagged LOXL2, HA-tagged L2Δ13 or the empty vector. **(B)** Immunoblotting detection of wild-type and homozygous L2Δ13-overexpressing mice (n = 4). **(C)** Aldolase enzyme activity analysis of SHEE cells overexpressing LOXL2 or L2Δ13 (left; n = 3), wild-type and L2Δ13-overexpressing mice (right; n = 4). Data show means ± SD, ****P* < 0.001. **(D)** Western blotting (left) and aldolase activity determination (right) of KYSE510 esophageal cancer cells upon deple tion of LOXL2. **(E)** KYSE510 cells were permeabilized with digitonin (30 μg/mL) for 5 min. Supernatant and cell lysate were subjected to immunoblotting. **(F)** Quantification of aldolase activity in the supernatant by immunoblotting of cells from **(E)**. Means ± SD, n = 3. ****P* < 0.001 by the *t*-test. **(G)** KYSE510 cells following depletion of LOXL2 were lysed and fractionated. Vimentin served as a marker for the cytoskeletal fraction (CF) and GAPDH for the soluble fraction (SF). Fractions from the cells transfected with scrambled shRNA are controls for the fractionation procedure.

Figure S7

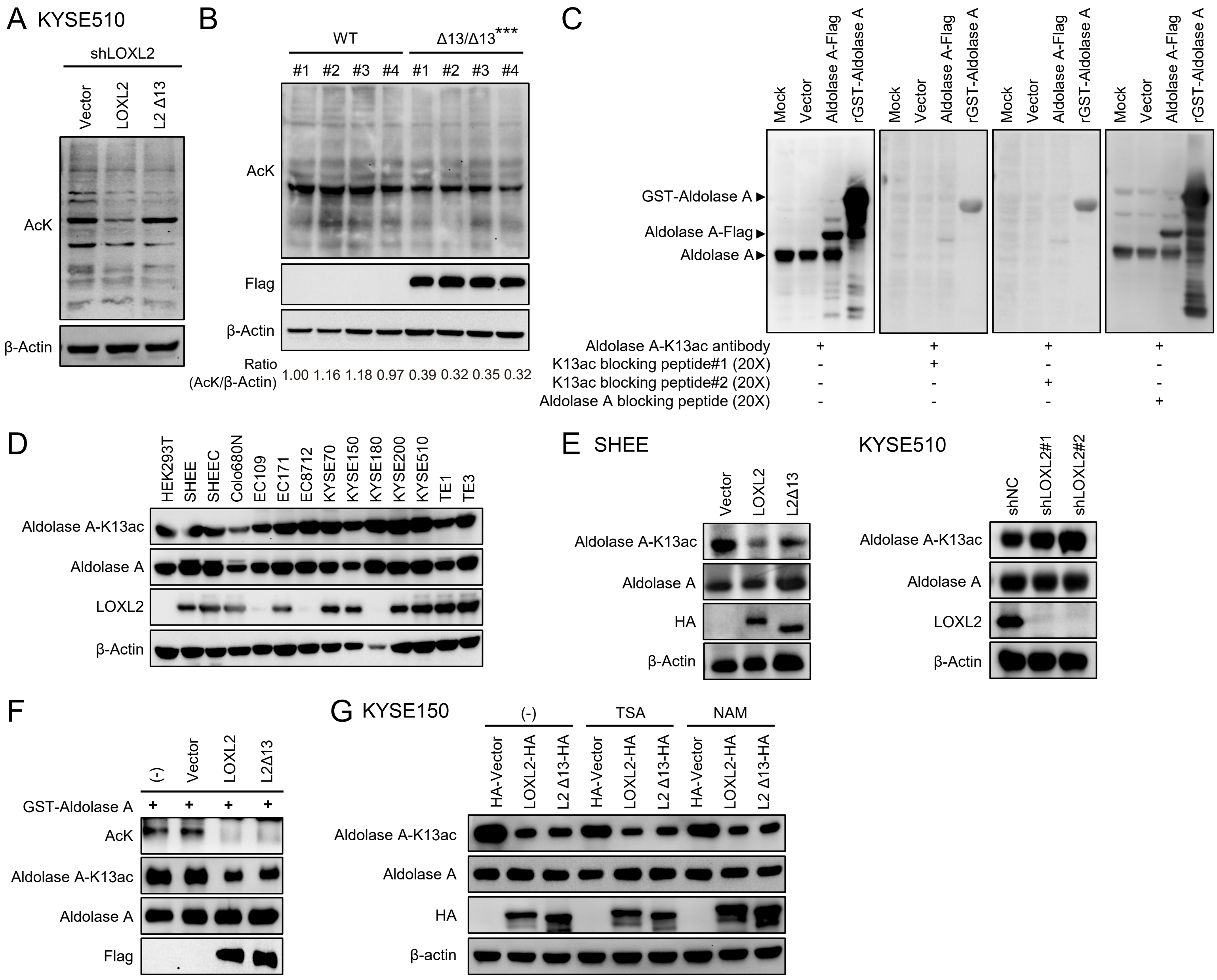

Figure S7. LOXL2 and L2Δ13 catalyze deacetylation of aldolase A-K13.

(A) Acetylation level of total proteins from whole cell lysates of stably LOXL2-silenced KYSE510 cells following LOXL2/L2Δ13 re-expression by Western blotting with anti -acetyl-Lys antibody. **(B)** Acetylation level of total proteins from livers of wild-type and L2Δ13-overexpressing mice. Ratios of AcK/β-actin between two groups were quantifiably analyzed by *t*-test, ****P* < 0.001. **(C)** Specificity of antibody directed against the acetylation of aldolase A-K13 was detected by Western blotting in untreated HEK293T cells, HEK293T cells expressing Flag-tagged aldolase A or Flag-tagged empty vector and purified recombinant GST-aldolase A from bacteria. Positive staining of aldolase A-K13ac was strongly blocked by indicated matching blocking peptides (#1 and #2) to the aldolase A-K13ac antibody, but not by the peptide to the total aldolase A antibody. **(D)** The expression levels of aldolase A-K13ac, total aldolase A and full-length LOXL2 in nonmalignant cells (HEK293T and SHEE) and different types of esophageal cancer cells. **(E)** Western blotting analysis of aldolase A acetylated at K13 (aldolase A-K13ac) and total aldolase A in SHEE cells expressing HA-tagged LOXL2 or HA-tagged L2Δ13 and KYSE510 cells silenced for LOXL2 expression. **(F)** Flag-tagged LOXL2, L2Δ13 and empty vector proteins were purified from HEK293T transfectants using Flag antibody, and then incubated with GST-aldolase A purified from bacteria in the LOXL2/L2Δ13 reaction buffer for in vitro deacetylase activity assay. **(G)** KYSE150 cells were transfected with the indicated plasmids and treated with or without traditional histone deacetylase inhibitors, including trichostatin A (TSA) and nicotinamide (NAM).

Figure S8

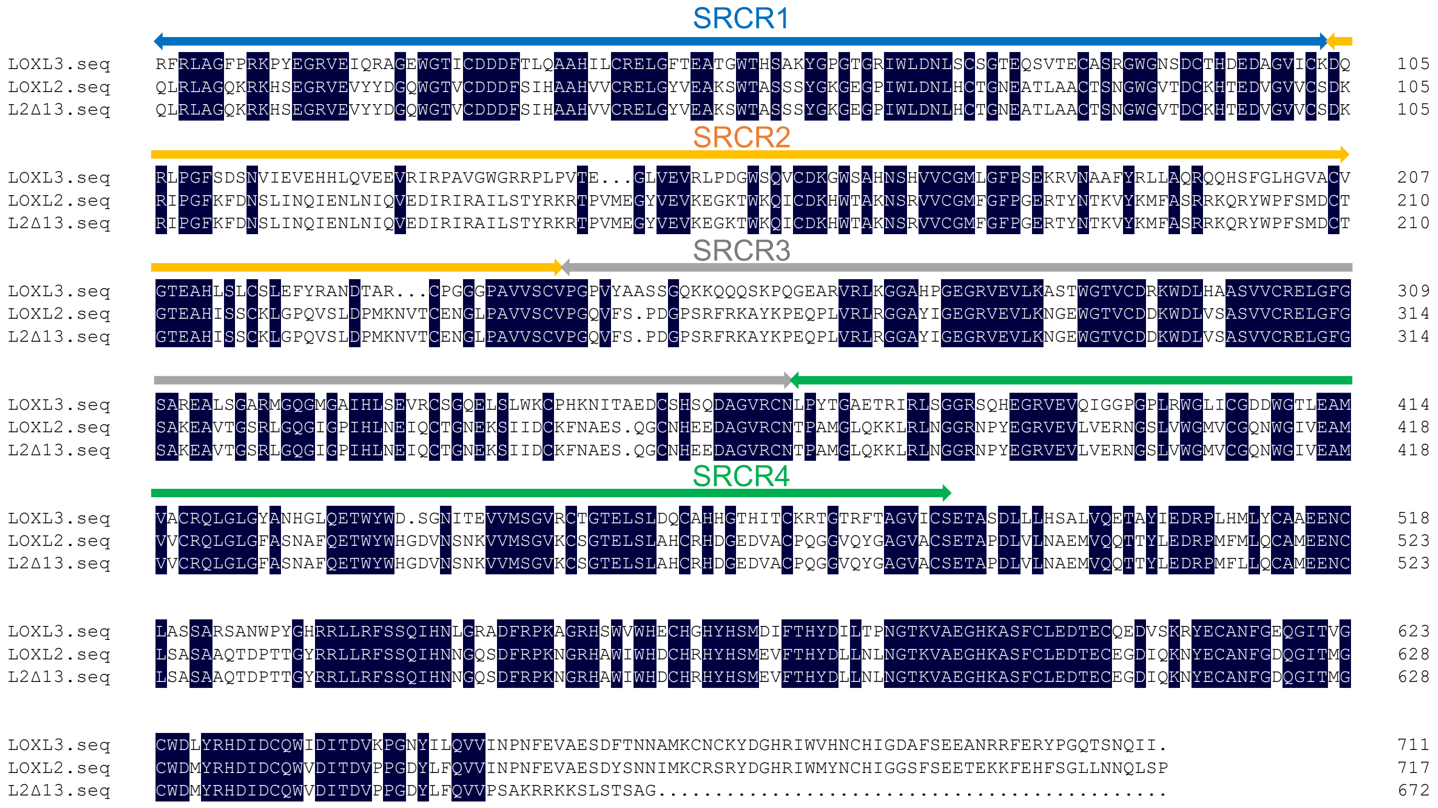

Figure S8. Sequence alignment among LOXL3, LOXL2 and L2Δ13.

Amino acid sequence alignment among LOXL3, LOXL2 and L2Δ13 for their N-terminal scavenger receptor cysteine-rich (SRCR) repeats and C-terminal domain.
